# Gut microbiota and synbiotic foods: Unveiling the relationship in COVID‐19 perspective

**DOI:** 10.1002/fsn3.3162

**Published:** 2023-01-11

**Authors:** Noor Akram, Farhan Saeed, Muhammad Afzaal, Yasir Abbas Shah, Aiza Qamar, Zargham Faisal, Samia Ghani, Huda Ateeq, Muhammad Nadeem Akhtar, Tabassum Tufail, Muzzamal Hussain, Aasma Asghar, Ammara Rasheed, Entessar Al Jbawi

**Affiliations:** ^1^ Department of Food and Nutrition Government College University Faisalabad Pakistan; ^2^ Department of Food Science Government College University Faisalabad Pakistan; ^3^ Department of Nutrition and Health Promotion University of Home Economics Lahore Lahore Pakistan; ^4^ Institute of Food Science and Nutrition Bahauddin Zakariya University Multan Multan Pakistan; ^5^ Faculty of Pharmaceutical Sciences Government College University Faisalabad Punjab Pakistan; ^6^ University Institute of Diet and Nutritional Sciences The University of Lahore Lahore Pakistan; ^7^ Agricultural Extension Directorate MAAR Damascus Syria

**Keywords:** COVID‐19, gut microbiota, immune system, nutrition, probiotics

## Abstract

The Coronavirus disease 2019 (COVID‐19) has spread across the globe and is causing widespread disaster. The impact of gut microbiota on lung disease has been widely documented. Diet, environment, and genetics all play a role in shaping the gut microbiota, which can influence the immune system. Improving the gut microbiota profile through customized diet, nutrition, and supplementation has been shown to boost immunity, which could be one of the preventative methods for reducing the impact of various diseases. Poor nutritional status is frequently linked to inflammation and oxidative stress, both of which can affect the immune system. This review emphasizes the necessity of maintaining an adequate level of important nutrients to effectively minimize inflammation and oxidative stress, moreover to strengthen the immune system during the COVID‐19 severity. Furthermore, the purpose of this review is to present information and viewpoints on the use of probiotics, prebiotics, and synbiotics as adjuvants for microbiota modification and its effects on COVID‐19 prevention and treatment.

## Graphical abstract

This review emphasizes the necessity of maintaining an adequate level of important nutrients to effectively minimize inflammation and oxidative stress, and also to strengthen the immune system during the COVID‐19 severity.

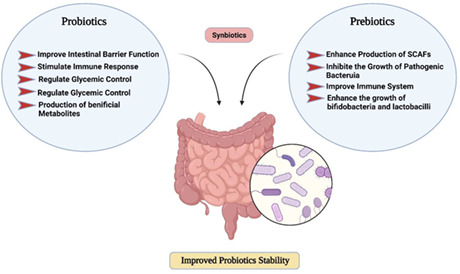

## 1 INTRODUCTION

The novel coronavirus has been causing a global health disaster since December 2019. COVID‐19 is an acute pulmonary health disease caused by “Severe Acute Respiratory Syndrome coronavirus‐2” (SARS‐Cov‐II virus). It has been reported that this virus had infected more than 210 million people and 4.4 million have died globally. Representative findings include high fever, coughing, sneezing, pneumonia, and loss of taste and smell (Huang et al., 2020). Moreover, 18% of COVID‐19 patients often suffer from gastrointestinal problems like diarrhea and vomiting (Cheung et al., 2020).

Some medical conditions and factors like age and sex, and other bad lifestyle factors such as smoking, exert an influence on the host metabolic status which determines the clinical severity of COVID‐19 (Kilgore, 2020). Some prevailing diseases come into view that can make worse the COVID‐19 infection, like hypertension, diabetes (type 2), cardiovascular diseases (CVD), cancers, and smoking, all of these factors regulate the illness course, progression, and outcome of COVID‐19 (Bae et al., 2021). The most diverse ecological community of microorganisms in the human body is “Gut microbiota.” It is another important factor that plays a condemnatory role during pulmonary infection. The human gastrointestinal tract possesses millions of microorganisms, and alterations in the compostition of gut microbiota due to different factors is termed “dysbiosis,” which has been interlinked with various human diseases. Some studies have used metagenomics sequencing proposed data regarding the clinical findings of COVID‐19 patients; the microbial diversity is decreased as compared to pathogens in fecal samples (Yeoh et al., 2021). Moreover, various beneficial microbes like *Eubacterium rectale* and *Faecalibacterium prausnitzii* have been reported to drain in COVID‐19 patient's fecal samples. However, opportunistic pathogens such as *Collinsella* species and *Morganella morganii* were exclusively high in fecal samples of SARS‐Cov‐II patients (Zuo et al., 2020). These studies have concluded that with an increase in pathogenic microorganisms is intently connected with sickness seriousness in SARS‐Cov‐II infection.

Foods having essential nutritional compounds have been very effective in the severity of SARS‐Cov‐II. Consumption of nutrients with antioxidant and antiinflammatory potential is beneficial for COVID‐19 patients as imprudent production of proinflammatory cytokines and acute inflammation takes place in SARS‐Cov‐II infection. Moreover, plant‐based phenols, omega‐3 fatty acids, zinc, vitamins, and polysaccharides are very major dietary nutrients in SARS‐Cov‐II infection (Iddir et al., 2020). Contrarily, acute changes occur in gut microbiota due to stress, injury, and inflammation, however, diet along with environmental factors can regulate the diversity of gut microbiota (Voigt et al., 2014). The gut microbiota has been demonstrated to be affected by a variety of dietary components; intake of proteins from peas and whey enhances gut beneficial bacteria like *Lactobacillus* and *Bifidobacterium*, on the other hand, whey has been reported to decrease *Bacteroides fragilis* and *Clostridium perfringens* pathogens in the gut (Earley et al., 2015). Essentially, it was noticed that diet low in fat leads to more fecal overflow of *Bifidobacterium*, while a diet high in saturated fat increases the abundance of *Faecalibacterium prausnitzii* (Dominika et al., 2011).

Moreover, a fiber‐rich diet encourages the development of healthy microbiota (Le Chatelier et al., 2013). Dietary fibers are considered as microbiota's most accessible carbohydrates which improve intestinal health and also provide energy to the host. However, prebiotics such as polydextrose, maize fiber, and insulin have been reported to boost gut diversity, immunity, digestion, and health, particularly in elderly people (Bouhnik et al., 2007). Gut microbiota and host immunity are correlated. Diet is again a predominant factor that regulates the host metabolism and elaborates the diversity and functions of gut microbiota (Hahn et al., 2017). Disturbances in nutritional status cause impairment of immune responses (De Santis et al., 2015). It is important to understand how the immune system responds to viral vaccines and how the efficacy of the vaccine can be increased. The efficacy of the vaccine is influenced by various factors like good nutrition, diet, sex, age, genetics, and immunity; these factors have great variation among low‐, middle‐, and high‐income settings (Valdez et al., 2014). Poor immune responsiveness to vaccination has been shown in low‐income countries and various factors are associated with this poor response status, such as genetics, beneficial microbiomes, environmental factors, and interlinked pathogens (Parker et al., 2014; Pulendran, 2014). Early‐life environmental factors that influence the microbiome such as the delivery method of birth, diet, and hygienic conditions can affect vaccine efficacy (Guaraldi & Salvatori, 2012). Moreover, micronutrient deficiencies are correlated with undernourishment and less diverse microbiota; particularly, in low‐ to middle‐income countries (LMICs), children are affected by vitamin A deficiency (VAD; Wirth et al., 2017). However, VAD remarkably affects vaccine efficacy and mucosal immunity (Hall et al., 2011).

Dietary components like dietary fiber can serve as prebiotics to enhance the abundance of probiotics in the gut. Moreover, plant‐based fibers increase the profusion of probiotics such as *Bifidobacterium* and *Lactobacillus*, while decreasing pathogenic bacteria like *Clostridium* (Iddir et al., 2020). The gut produces SCFAs such as propionate, acetate, and butyrate when certain prebiotic dietary fibers are degraded by gut microbes. SCFAs are major immunomodulatory metabolites that can enhance the responsive activities of CD8+ T‐cells and B‐cells in the immune system (Alameddine et al., 2019; Trompette et al., 2018). Evidence from recent studies has proved that lung infections are severely affected by less food consumption ultimately leading to alteration in the gut microbiome, as the severity of viral infections is correlated with food intake and gut microbiota diversity. It is needed to understand that gut microbiota is very essential in maintaining good health and lessening the severity of disease or viral infections. The gut microbiomes generate metabolites that can systematically change immune responses. The gut microbiota diversity is directly correlated with the host immune system (Groves et al., 2020).

**FIGURE 1:**
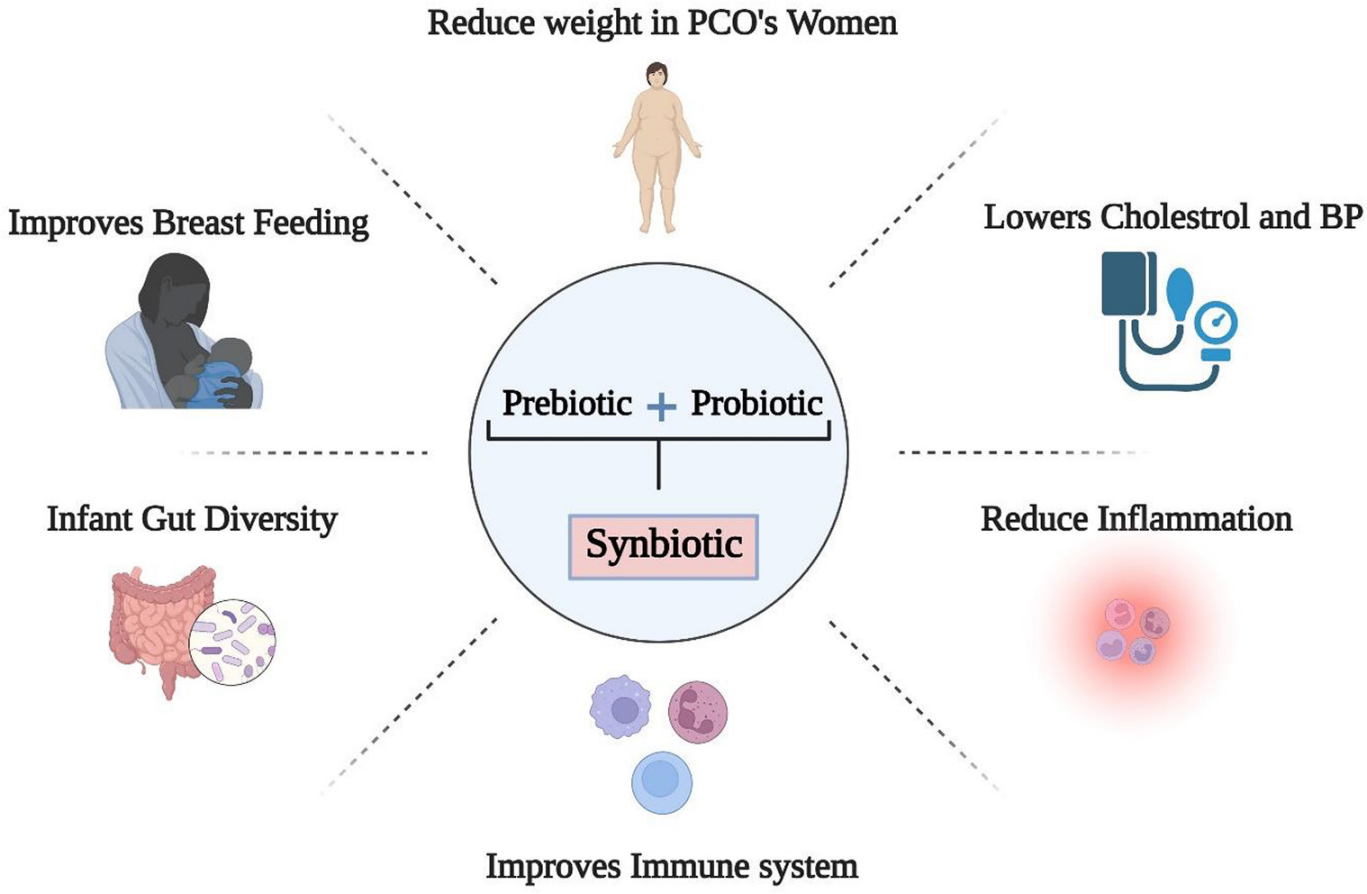
Adjuvant effect of prebiotics and probiotics on human health

Several bacteria are found in the human respiratory system, and internalization of respiratory viruses occurs mostly through this route. Probiotics are another friendly group of bacteria that, when swallowed or supplied in a certain concentration, have a good impact on human health. Probiotic strains like *Bifidobacterium* and *Lactobacillus* can trap viruses and interfere with their binding to host cell receptors, which is advantageous to the host's health (Kanauchi et al., 2018). Results from various studies have shown that probiotics may be useful in the treatment of viral infections such as COVID‐19, viral infections, pulmonary tract infections, cystic fibrosis, chronic inflammatory diseases, PCOs, weight loss, and improving lactation in women (Alberca et al., 2021; Bajinka et al., 2022; Robichaud et al., 2021). Consumption of probiotic‐rich meals or supplements has been proven to impact immune function by modifying the microbiota's endogenous metabolic activity (Galdeano et al., 2019). Moreover, the gut microbiota is a complex collection of microorganisms that colonize the mucosal surfaces of the gut. These bacteria are considered key elements in health due to their significant metabolites, immune system control, and defense of the body against infections (Human Microbiome Project Consortium, 2012). The effects of prebiotics and probiotics on human health are shown in Figure 1.

## 2 MAINTAINING A HEALTHY IMMUNE SYSTEM DURING COVID‐19 CRISES

World Health Organization had reported COVID‐19 as a health emergency. Challenges emerge to resist the severity of infection ideally and enhance immunity. The role of food as a natural source of immunomodulation cannot be overlooked throughout the vaccination campaign. It has been reported that nutrients like probiotics, prebiotics, folic acid, zinc, and vitamins C, D, E, and A are prime modulators for a healthy immune system (Saeed et al., 2016). As an effective immune response depends upon a balanced diet and accurate nourishment of gut microbiota, it is reported that adequate protein intake is essential for antibody production. Micronutrient deficiencies are associated with high‐risk infections and the severity of diseases, particularly vitamin A and zinc (Iddir et al., 2020). However, oxidative stress and inflammation extremely regulate the responses of the immune system (Lauridsen et al., 2019). Oxidative stress and inflammation have been shown a great impact on the immune system. Moreover, vitamins like A, D, and C along with some plant‐based proteins like polyphenols, carotenoids, and flavonoids have antiinflammatory and antioxidant potential; these dietary nutrients are vital for a strong and healthy immune system (Iddir et al., 2020). Furthermore, vaccination to combat COVID‐19 is now available, and the relevance of natural forms of inhibition and treatment cannot be overlooked. In this regard, food and dietary habits are important factors in determining overall health and resistance (Lindgren et al., 2018).

### 2.1 Proteins

According to the Institute of Medicine, the USA recommended daily allowance (RDA) for protein 0.8/kg body weight. Increased risk of infection has been recognized in low‐income countries as they are protein malnourished due to low protein availability (Rodríguez et al., 2011). Immunoglobulins and lymphoid tissues associated with the gut play important role in defense against infection; these components are interlinked with protein availability (Amaral et al., 2006). Moreover, studies have reported that very low protein intake decreases the antibodies abundance and increases the risk of viral infections (Taylor et al., 2013). Protein intake of high biological value and essential amino acids may lower inflammation during infection and improve satiety (Chungchunlam et al., 2017). High‐quality proteins have proven primary component of the antiinflammatory diet (O'Keefe et al., 2008). Branched‐chain amino acids enhance the gut barrier by increasing the immunoglobulin level in the intestine (Ren et al., 2015).

### 2.2 Lipids

The number and type of lipids in the diet have an impact on the composition and function of the gut microbiota. Therefore, dietary lipids can have an impact on host physiology via interacting with the microbiota in the gut. Lipids have an impact on the gut microbiota both as substrates for bacterial metabolic processes and by suppressing bacterial development (Schoeler & Caesar, 2019). Recent studies indicate that cellular metabolism, including lipid metabolism, affects macrophage activity. Lipids provide energy to macrophages and serve as the building blocks for bioactive lipids and cellular membranes. During macrophage activation, lipids also control signaling and gene regulation (Yan & Horng, 2020).

However, antiinflammatory effects contribute to a healthy immune system. Fatty acids can remarkably change immune responses. Particularly, fatty acids (FAs) regulate the immune cell functioning such as macrophages, neutrophils, innate lymphoid cells, T‐cells, and B‐cells (Radzikowska et al., 2019). Two essential fatty acids omega‐3 and omega‐6 cannot be produced by the human body. Both these FAs are the most potent antiinflammatory nutrients and must be taken through diet (Innes & Calder, 2018; Serhan & Levy, 2018).

### 2.3 Carbohydrates

The role of gut microbiota in the immune system is vital and its modulation by adequate nutrition affects the severity of viral infection, but unfortunately, studies regarding, diet, gut, and infection are few (Dubourg et al., 2019). Dietary fibers are complex carbohydrates that play a major role in inflammation (Galland, 2010). Various kinds of dietary fiber are not the sole forces for stomach or gut well‐being, but they can help in the production of SCFA that nourishes gut microbiota spices. SCFAs level is regulated by diet and plays an important role in immunomodulatory function (Parada Venegas et al., 2019). Moreover, foods with a high glycemic index such as processed foods have been reported to cause increased production of free radicals, which ultimately lead to oxidative stress and inflammation (O'Keefe et al., 2008). Contrarily, the consumption of dietary fiber is linked to a decreased mortality rate because of respiratory and infectious diseases in adult Americans (Park et al., 2011).

### 2.4 Minor nutritional components

Vitamin A is associated with the development of solid bodily fluid layers or mucous, particularly, for the respiratory and the digestive tract needed for mucin secretion and upgrading antigen levels (Watchorn et al., 2022). Globally, vitamin A is the most common micronutrient deficiency specifically in low‐ and middle‐income countries where meat intake is low (Ross, 2010). Primarily, a deficiency of vitamin A is linked with an increased probability of infection (Huang et al., 2018). Active forms of vitamin A are retinal, retinol, and retinoic acid, however, retinoic acid among them plays a major role in the maturation, differentiation, regulation, and functioning of the innate immune system (Shrestha et al., 2017). Vitamin A boosts tissue antigen immunity by boosting mucin production in both the gut and respiratory system (Huang et al., 2018). As a result, it is predictable that vitamin A deficiency has been linked to impaired vision. Neutrophils, macrophages, and B‐ and T‐cells all play a role in the maintenance of a healthy immune system (Singh et al., 2001). Moreover, it increases the levels of immunoglobulin A, G, and M in the blood and improves the functioning of the innate immune system (Huang et al., 2020). Furthermore, people with low vitamin A levels have morphological alterations in their epithelial and parenchyma cells lungs, which increases the risk of lung illness and dysfunction (Timoneda et al., 2018). This is critically significant in terms of COVID‐19's impact on pulmonary function (Siddiqi & Mehra, 2020).

Vitamin D is obtained through eggs, fortified milk, fish, and mushrooms in the diet. It may also be produced under the skin in the presence of UV light from cholesterol (Mosekilde, 2005). When it comes to the immune system, vitamin D has been shown to modulate both the innate and adaptive immune systems (Childs et al., 2020). The active form of vitamin D is calcitriol (1,25 dihydroxy vitamin D), synthesized by the hydroxylation mechanism in the liver and kidney that is involved in calcium homeostasis, and ultimately, enhances bone health; however, it has also been proven to influence immune system (Mosekilde, 2005). The function of the T‐cell is strongly connected to vitamin D status (von Essen et al., 2010). Toll‐like receptors (TLR) in the innate system let macrophages recognize lipopolysaccharide (LPS), which acts as a replacement for bacterial immunity (Kaminogawa & Nanno, 2004).

A meta‐analysis had shown that vitamin D therapy remarkably improves the severity of chronic obstructive pulmonary disease (COPD) (Li et al., 2020). Another analysis revealed that there was a lower incidence of COVID‐19 and influenza infections along with death (Grant et al., 2020). A recent retrospective analysis of confirmed cases of SARS‐CoV‐2 infection had shown that low vitamin D levels were significantly linked to a higher risk of mortality, particularly, in geriatric and male individuals with prior illnesses (Raharusun et al., 2020).

High intake of ascorbic acid or vitamin C has been linked to lower C‐reactive protein (Wannamethee et al., 2006). Vitamin C is essential for immune system function, particularly immune cell activity; it is acknowledged as a traditional antioxidant that quenches free radicals immediately and prevents proinflammatory diseases. This vitamin boosts phagocytosis, boosts oxidant production, boosts microbial death, and speeds up neutrophil transit to the infection site. Its role as a cofactor for various gene regulatory and biosynthetic enzymes is thought to have a role in immunological modulation (Carr & Maggini, 2017). Vitamin C has been demonstrated to increase neutrophil migration to the infection site, triggering the production of reactive oxygen species (ROS) and phagocytosis (Carr & Maggini, 2017; Carr & Melcher, 2017). A clinical trial among 50 COVID cases was performed, in which high‐dose vitamin C intravenous intervention was given and outcomes have shown positive changes in the oxygenation index of COVID patients (Cheng, 2020). Adequate levels of vitamin C have been proposed as a therapy treatment for those suffering from the common cold and pneumonia (Hemilä, 2017).

Zinc is a trace element that the human body cannot produce or store, thus it must be obtained via food (Stefanidou et al., 2006). Zinc inadequacy is a severe issue in public health that affects people globally (Wessells & Brown, 2012). Individuals with zinc deficiency are more prone to viral infections (Read et al., 2019). Zinc is also thought to be essential for T‐cell receptors that need the intracellular binding of tyrosine kinase, which is necessary for their growth and survival as well as activation of T‐lymphocyte activation (Wintergerst et al., 2006). Zinc plays a crucial role in cytokine release and triggering CD8+ T‐cell proliferation, as well as immune cell development and differentiation. It also helps to modify cytokine release and induce CD8+ T‐cell proliferation (Wintergerst et al., 2007). Zinc enhances the production of macrophages and phagocytosis, elevates cytokine levels, promotes B‐ and T‐cells growth, and also inhibits free radicals‐induced injury (Wessels et al., 2017). In a recent study, the impact of poor zinc status on the elderly and its link to pneumonia were emphasized (Barnett et al., 2010). Essential nutrients in the therapy of COVID‐19 and other infections are presented in Table 1.

**TABLE 1:** Essential nutrients in the therapy of Covid‐19 and other infections

Nutrient	Study	Outcomes	References
Vitamin D	In Indonesia, two cohort studies of 780 patients with proven SARS‐CoV‐2 infection were performed. COVID‐19 mortality patterns with a special focus on vitamin D deficiency and related variables were investigated in this study	COVID‐19 mortality is highly linked to vitamin D deficiency	Raharusun et al. (2020)
Folic acid	The study proposed that pregnant women have a less chance of acquiring infection, particularly SARS‐Cov‐II, because of FA supplementation	Pregnant women with low folate RBC concentrations who do not take FA supplements had a higher chance of hospitalization. FA supplementation may protect against SARS‐CoV‐2, according to this research	Acosta‐Elias and Espinosa‐Tanguma (2020)
Ascorbic Acid	A preliminary study reveals the intravenous effects of vitamin C in patients with sepsis and ARDS, as well as molecular indicators of inflammation and vascular damage. In this study, vitamin C intravenous infusions were given to patients randomly every 6 h for 96 h	During infection, ascorbic acid (AA) alters ROS production, consequently, prevents cell death	Truwit et al. (2019)
Zinc	In vitro study was performed through the inhibition of RNA‐dependent RNA polymerase (RdRp) and 3C‐like viral proteinase (3CLpro)	Zinc (Zn) inhibits SARS‐CoV‐2 replication and regulates IL‐1β and TNF‐α levels and also modulates cytokine storm in COVID‐19	Pormohammad et al. (2021)

## 3 VACCINE EFFICACY OF COVID‐19

The immunological response to the SARS‐CoV‐2 vaccine and the vaccine's side effects are likely to be influenced by the gut microbiome. According to preliminary results of an ongoing clinical investigation, significant changes were observed in subjects who took a probiotic formula for 2 months and had greater blood SARS‐CoV‐2 IgG antibody levels and lower proinflammatory cytokines. Large public‐based research in the United States enlisted more than 10,000 people to figure out the link between COVID‐19 vaccination effectiveness and gut microbiota. The potential role of gut microbiota in protection against SARS‐CoV‐2 infection is close to the bottom (Lau & Yu, 2022). In children living in low‐income and middle‐income countries, undernourishment and repetitive gastrointestinal infections are associated with the failure of oral vaccines. Mechanistic studies of the relationship among the microbiome, host genetics, and viral infections are needed to understand how immune responses to viral vaccines can be modulated (Vlasova et al., 2019).

It is currently unclear to what extent SARS‐CoV‐2 vaccinations provide universal protection, and scientists are working to find out. Researchers from all around the world are seeking to find the optimal quantity of self‐antigens or immunological markers. The efficacy of the COVID‐19 vaccination is tightly linked. The increase in the number of instances caused by the delta variation throughout the world has encouraged several governments to think about booster vaccinations for at‐risk populations regardless of unsubstantiated arguments (Callaway, 2021). In 2019, the gut microbiome was shown to alter vaccine effectiveness after healthy participants taking antibiotics before the H1N1 influenza vaccination showed a substantial reduction in antibody response (Hagan et al., 2019).

## 4 GUT–LUNG AXIS AND POTENTIAL ROLE OF THE GUT IN IMMUNITY

When the virus (SARS‐CoV‐2) enters host cells, the interface between innate and adaptive immunity is rapidly activated (Chowdhury et al., 2020). Trillions of microorganisms live in the human gut microbiota, including viruses, archaea, bacteria, and fungi (Gill et al., 2006). *Bacteroidetes*, *Proteobacteria*, *Actinobacteria*, and *Firmicutes* are the major four phyla that are dominant in the gut of healthy individuals (Villanueva‐Millán et al., 2015). There is a gut–lung axis that facilitates intense communication between the two organs, including microbial and immunological interactions (Enaud et al., 2020). The host provides the bacteria home and food and the microorganisms support the host by providing protective immunity against viral infections and also regulating various physiological activities such as nutritional digestion. A variety of disorders is considered with less diverse gut microbiota (Mosca et al., 2016). Moreover, premature birth, cesarean section, poor breastfeeding, and antibiotic therapy, alone or in combination, are all linked to inefficient microbiota development. During infancy, immunological problems or inflammatory illnesses are common. Changes in the gut microbiota, often known as “gut dysbiosis,” have been linked to a variety of diseases and illnesses, including type 2 diabetes, IBD, cardiovascular disease, and depression (Dhar & Mohanty, 2020). Several lung disorders, including allergies, asthma, and cystic fibrosis, have been linked to dysbiosis in the gut microbiota (Anand & Mande, 2018). Despite increased food consumption, deficiency in specific nutrients is typically noted in overweight and obese individuals due to an imbalanced diet (Zhang, Liu, et al., 2020). The microbiota may have a key role in the immune response to bacterial infections in the lungs, but the mechanisms behind this need to be researched further (Enaud et al., 2020).

“Gut‐lung axis” is a critical cross‐talk between the gut microbiota and lungs; gut microbiota is found to influence lung health through this link (Keely et al., 2012). This cross‐talk is considered to be bidirectional and endotoxins, and microbial metabolites may influence the lungs causing inflammation that can also affect the gut microbiota (Dumas et al., 2018). Bacteria from the groups *Prevotellaceae* and *Ruminococcaceae*, *Bacteroidaceae*, *Lachnospiraceae*, and *Rikenellaceae* are abundant in the colon (Hall et al., 2017). Numerous pieces of evidence imply the presence of different bacteria in the lungs, similar to the gut microbiota (Bingula et al., 2017). *Bacteroidetes*, *Proteobacteria*, and *Firmicutes* are dominant in the lungs, whereas *Firmicutes* and *Bacteroidetes* dominate in the gut (Zhang, Zhu, et al., 2020). The dysbiosis microbiome may trigger an inflammatory response that the coronavirus can take full advantage of. The dysbiosis microbiome may trigger an inflammatory response that the coronavirus can take full advantage of. When a coronavirus infects the gut, inflammatory substances called cytokines are increased by additional cytokines. The resulting inflammation might set off a “cytokine storm,” a rogue immune response that can do more harm than the virus itself, causing multiorgan impairment (Yang et al., 2020).

Respiratory virus infections are exceedingly prevalent, but little is known about how they affect the makeup and function of the gut microbiota. After a viral lung infection, a study had shown considerable alteration in the gut microbiota. Weight loss during RSV or influenza virus infection was due to reduced food consumption (Groves et al., 2020). This offers the intriguing prospect that new SARS‐Cov2 might affect the gut microbiome as well. Alteration in gut microbiota has been linked to respiratory infections in multiple studies (Groves et al., 2020). The lung microbiota becomes abundant in bacteria from the gut in ARDS, most likely due to a hyperpermeable gut that allows germs to pass past the colon wall and into the lungs (Fanos et al., 2020).

**FIGURE 2:**
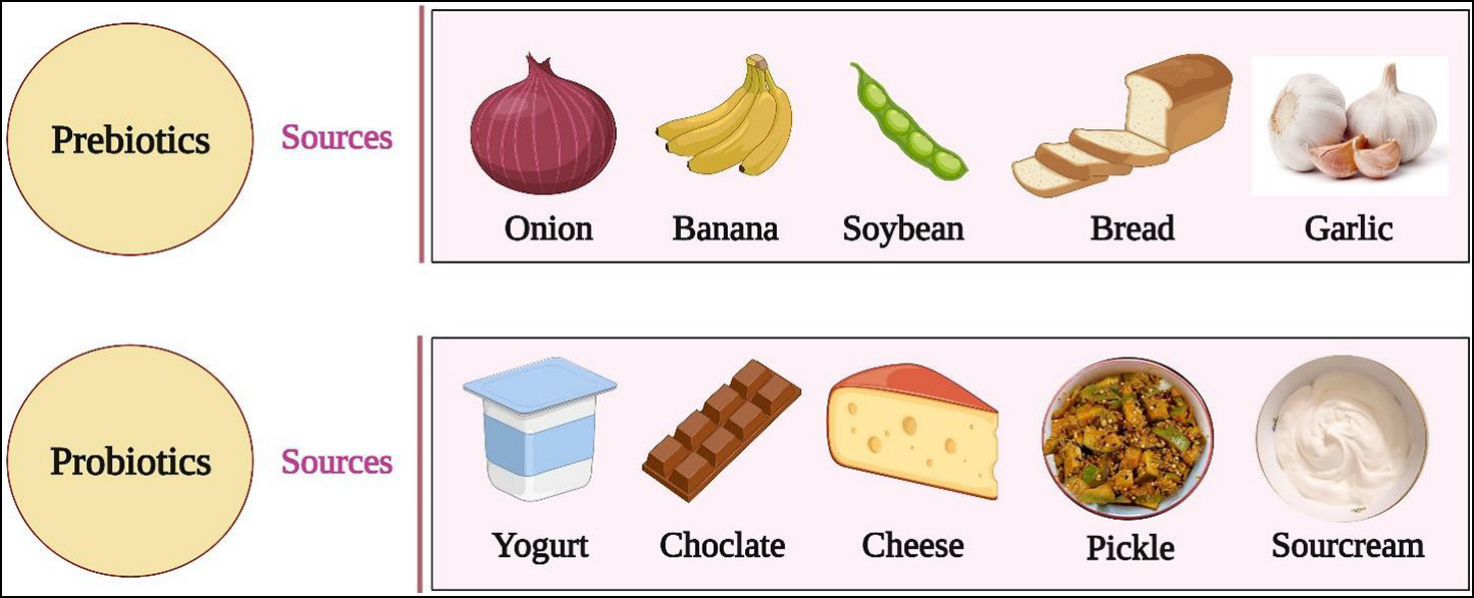
Sources of prebiotics and probiotics

Two significant clinical symptoms of COVID‐19 are acute respiratory distress syndrome (ARDS) and pneumonia, particularly, in older individuals and patients with a weak immune system (Lake, 2020). Elderly individuals with a weak immune system have shown substantial negative clinical manifestations. Gut microbiota interactions with the host are various, intricate, and bidirectional. The gut is thought to have a key role in the development and function of adaptive and innate immune systems (Negi et al., 2019). However, elderly adults have less diversified microbiota and beneficial microbes such as *Bifidobacterium* are deficient in the gut (Nagpal et al., 2018). Improving gut microbiota could be one of the prophylactic ways by which the impact of this disease can be minimized in old people and immune‐compromised patients (Dhar & Mohanty, 2020). The gut microbiota and immunological homeostasis appear to have a strong correlation. Antimicrobial peptides are produced in the gut when beneficial microbes compete for nutrients and habitat (Moens & Veldhoen, 2012). Furthermore, the derived signals from gut microbiota adjust immune cells for pro‐ and antiinflammatory responses to enhance immunity, which may reduce disease susceptibility and progression (Negi et al., 2019). Furthermore, it has been shown that the gut and lungs share a mucosal immune system and that the gut–lung axis links the inflammatory process and immunological responses (Antunes et al., 2020). Different sources of prebiotics and probiotics are shown in Figure 2.

## 5 IMPACT OF PROBIOTICS AND SYNBIOTIC ON THE SEVERITY OF COVID‐19

Probiotics are living microorganisms that provide a health benefit to the host when given in sufficient concentrations (Hill et al., 2014). The majority of *Lactobacillus*, *Bifidobacterium*, *Saccharomyces*, and *Bacillus* are the most often utilized probiotics in supplements, while *Lactobacillus* is the most generally used. *Bifidobacterium* and *Lactobacillus* acidophilus are solely utilized in foods (Antunes et al., 2020).

Probiotics, in combination with a tailored diet, may help to balance the microbiota and boost the host's immune system function. Probiotics may be able to moderate amplified immunological responses, such as those caused by antibiotics and the cytokine storm caused by COVID‐19. Probiotics may decrease local and systemic inflammatory reactions in COVID‐19 by minimizing the cytokine storm on the axis of the stomach, lungs, and brain (Ailioaie & Litscher, 2021). Modern therapeutic approaches have been substantially influenced by the interaction between the GM and host immune system, and altering the gut microbiota (GM) to improve the acquired immunological response in aged people is drawing interest. Diversification of gut microbiota with probiotics, prebiotics, or postbiotics may be an effective method to improve the effects of immunization in immune‐compromised elderly people to boost immunological responses, including long‐term NK‐cell activity and antibody titers, as well as restore the GM balance (Akatsu, 2021).

The gut microbiota also has a significant impact on immunity and inflammation. It also has an effect on pulmonary function via the gut–lung axis. The role of the host microbiome in infection and pathogenicity has recently been reported. Understanding the microbiomes of the stomach and lungs might lead to novel medicinal methods (Allali et al., 2021). Nutritional techniques to boost immunity against SARS‐CoV‐2 are being considered as most promising techniques by various studies. Prebiotics, on the other hand, may improve gut immunity by boosting particular gut microorganisms selectively. Probiotics, prebiotics, and fermented foods have been shown to improve gut and pulmonary health along with immunity at various levels of proof. Without a guarantee of efficacy against COVID‐19, the inclusion of such nutritional foods in the diet may reduce gut inflammation, reduce the intensity of infection, and boost mucosal immunity (Antunes et al., 2020).

COVID‐19 has been demonstrated to have impacts on gut microbiota dysbiosis, and probiotics have shown a remarkable role in the treatment of pulmonary tract viral infections. These findings may boost the impact of probiotics as a treatment therapy for COVID‐19 (Bottari et al., 2021). Oral probiotics, which are beneficial living bacteria that can give medicinal benefits to the host when provided in safe doses, may be worth examining as a potential preventative or therapeutic method to minimize coronavirus illness and the intensity of the symptoms (Baindara et al., 2021). The effects of probiotic and symbiotic supplementation on human health are presented in Table 2.

**TABLE 2:** Effects of probiotic and symbiotic supplementation on human health

Supplement	Bacterial strain	Subject	Outcomes	References
Probiotic	*Bifidobacterium longum*	Adults	High niche difference was observed and vacant niches were available when there were no competitors for externally added microbes	Maldonado‐Gómez et al. (2016)
Probiotics	*Bifidobacterium infantis*	Infants	EVC001 had a clear advantage due to the presence of HMOs in breastmilk, and its administration along with breastfeeding encourages optimum conditions for a niche. EVC001 prevents the growth of gram‐positive bacteria by lowering the pH, moreover HMOs encourage bacterial strain to persist in the infant gut	O'Brien et al. (2021) (Frese et al., 2017)
Probiotics	*L. rhamnosus* and *B. animalis*	Adults	During probiotic treatment, improvement in gut diversity was observed in 13% of patients with cystic fibrosis disease	Van Biervliet et al. (2018)
Probiotics and Insulin	*Lactobacillus rhamnosus*	Adults	Gut dysbiosis may reduce depression and chronic inflammation. The probiotic strain had shown antiinflammatory and antidepressant effects; particularly, cosupplementation with insulin in CAD patients had shown beneficial impacts	Bohlouli et al. (2021)
Probiotics	*Lactobacillus acidophilus* *Lactobacillus casei* *Bifidobacterium bifidum*	Women	Remarkable effects had been observed on glycemia, weight loss, and lipid profile of women with polycystic syndrome by probiotic strains supplementation	Ahmadi et al. (2017)
Probiotic	*L. acidophilus* *L. reuteri* *L. fermentum* *B. bifidum*	Women	Probiotic supplementation significantly improves weight loss, cholesterol, insulin, and hormonal balance in PCOs patients	Tabrizi et al. (2022)
Probiotic	*Lactobacillus pentosus*	Female BALB/c mice	Remarkable NK‐cell activity was observed along with higher expression of IL‐12 and IFN in lungs, and the endurance rate was high as compared to viral load in lungs with respect to influenza virus infection	Izumo et al. (2010)
Probiotic	*Lactobacillus rhamnosus*	Female BALB/c mice	Infected mice's clinical symptoms were relieved in response to influenza virus, and the viral burden in their lungs is dramatically reduced	Kawase et al. (2010)
Probiotic	*Lactobacillus rhamnosus*	6269 participants	According to meta‐analysis of 23 studies, probiotics decrease the severity of pulmonary tract infections and improve mortality rate	Wang et al. (2016)
Probiotic	*Lactobacillus casei*	Adult/Children	Probiotics were found to be useful in the treatment of antibiotic‐associated diarrhea	Dietrich et al. (2014)
Probiotic	*Lactobacillus rhamnosus*	Participants	A meta‐analysis of 52 trials had shown that probiotics are effective against multidiseases like acute respiratory tract infections, antibiotic‐associated diarrhea, acute infectious diarrhea, baby colic, and necrotizing enterocolitis	Liu et al. (2018)
Probiotic	*Lactobacillus gasseri*	Female BALB/c mice	Decreased expressions for proinflammatory cytokines, reduced weight loss, and viral load in the lungs of infected mice were observed	Eguchi et al. (2019)
Probiotic	*Lactobacillus rhamnosus*	Participants	A meta‐analysis of 17 trials had proved that antibiotic use and the incidence of common acute illnesses were both dramatically decreased when probiotic strains were used	King et al. (2019)
Synbiotic	Combined strains	5 trials, 1049 Participants	A meta‐analysis implies that synbiotic intervention can help individuals with metabolic syndrome reduce their cardiometabolic risk factors. This study had shown that how a synbiotic intervention affected people's lipid profiles, insulin resistance, blood pressure, anthropometric measurements, and inflammatory markers	Piper et al. (2022)
Synbiotic Probiotic Prebiotic	Strains of genera used: *Lactobacillus* *Bifidobacterium* *Propionibacterium* *Streptococcus*	Pregnant and lactating women	The impact of symbiotic formulas was observed with the combination of probiotic strains and galacto‐oligosaccharides, fructo‐oligosaccharides, or bovine milk‐derived oligosaccharides Beneficial effects were observed in vaginally delivered, cesarean‐delivered, and breastfed newborns	Hertz et al. (2022)
Probiotic	*Streptococcus thermophiles* *Lactobacillus acidophilus* *Lactobacillus helveticus* *Lacticaseibacillus paracasei* *Lactiplantibacillus plantarum* *Levilactobacillus brevis* *Bifidobacterium lactis* *Bifidobacterium lactis* *Lacticaseibacillus rhamnosus*	Hospitalized Covid‐19 patients	An intervention of 2.4 billion CFU bacteria was given orally to 70 hospitalized Covid‐19 patients. The clinical circumstances of COVID‐19 patients improved significantly after a probiotic intervention	d'Ettorre et al. (2020)
Probiotic	Multiple strains	Covid‐19 cases	A retrospective study was conducted on 800 positive Covid‐19 cases to check the remission of diarrhea in Covid‐19 patients. In comparison to the placebo group, the duration of diarrhea in the probiotic group was much shorter. Individuals' gastrointestinal symptoms such as abdominal distension, nausea, vomiting, and others were reduced as a result of the various strains	Xavier‐Santos et al. (2022)
Probiotic	*Ligilactobacillus salivarius*	Covid‐19‐positive cases	29 COVID‐positive residents of a nursing home were intervened daily with probiotic strain per unit of 125 g. The study proposed that certain immunological variables might be used as nasal or fecal biomarkers to assess the advantages of a probiotic strain supplementation in the diet of elderly patients infected with SARS‐CoV‐2	Mozota et al. (2021)

## 6 CONCLUSION

The gut microbiota is widely known for providing a variety of health benefits, including disease protection. Although gut dysbiosis, which can be induced by a variety of factors, is linked to severe COVID‐19, it is essential to address the approaches to maintain the health of gut microbiota. Diet affects the makeup and functional capabilities of the gut microbiota, which in turn affects host biochemical processes, forming a system of mutual interaction and interdependence. Macronutrients, fiber, polyphenols, and prebiotics are all important factors in shaping the composition of gut microbiota. COVID‐19 is less severe when the gut microbiota is healthy, diverse, and abundant, as well it also increases the efficacy of vaccinations against COVID‐19.

## CONFLICT OF INTEREST

The authors declare that they have no conflict of interest.

## ETHICS STATEMENT

This article does not contain any studies with human participants or animals performed by any of the authors.

## CONSENT TO PARTICIPATE

Corresponding and all the coauthors are willing to participate in this manuscript.

## CONSENT FOR PUBLICATION

All authors are willing to the publication of this manuscript.

## Data Availability

Even though adequate data have been given in the form of tables and figures, all authors declare that if more data are required, then the data will be provided on a request basis.
